# Simultaneous RP-HPLC Estimation of Cefpodoxime Proxetil and Clavulanic Acid in Tablets

**DOI:** 10.4103/0250-474X.51945

**Published:** 2009

**Authors:** S. Malathi, R. N. Dubey, R. Venkatnarayanan

**Affiliations:** Department of Pharmaceutical Analysis, RVS College of Pharmaceutical Sciences, Sulur, Coimbatore-641 402, India

**Keywords:** Cefpodoxime proxetil, clavulanic acid, RP-HPLC, isocratic

## Abstract

A new, simple, precise, rapid and accurate RP-HPLC method has been developed for the simultaneous estimation of cefpodoxime proxetil and clavulanic acid from pharmaceutical dosage forms. The method was carried out on a Zorbax Eclipse XDB 5 μ C 18 (150×4.6 mm) column with a mobile phase consisting of acetonitrile:50 mM potassium dihydrogen phosphate buffer (pH 3.0, 70:30 v/v) at a flow rate of 1.0 ml/min. Detection was carried out at 228 nm. Aspirin was used as an internal standard. The retention time of clavulanic acid, cefpodoxime proxetil and aspirin was 4.43, 6.44 and 5.6 min, respectively. The developed method was validated in terms of accuracy, precision, linearity, limit of detection, limit of quantification and solution stability. The proposed method can be used for the estimation of these drugs in combined dosage forms.

Cefpodoxime proxetil, chemically [(RS)-1 (isopropoxy carbonyloxy)ethyl(+)–(6R,7R)-7[2-(2-amino-4-thiazolyl)-2(Z)methoxyiminoacetamido]-3-methoxymethyl-8-oxo-5-thia-1-azabicyclo [4.2.0.]oct-2-ene-2-carboxylate], is a third generation cephalosporin antibiotic having activity against gram positive and gram negative micro organisms. Clavulanic acid is chemically [2-R-(2α-3z,5α)]-3-(2-hydroxyethylidine)-7-oxo-1-azabicyclo[3.2.0]heptane-2-carboxylic acid. It is used as a beta lactamase inhibitor, enhances the activity of penicillin and cephalosporin antibacterials against many resistant strains of bacteria. Many methods have been described in the literature for the determination of cefpodoxime proxetil and clavulanic acid individually and in combination with other drugs[1–6]. However, there is no HPLC method reported for the simultaneous estimation of these drugs in combined dosage forms. Fixed dose combination containing cefpodoxime proxetil (100 mg) and clavulanic acid (28.5 mg) is available in the tablet form in the market. The aim of this work was to develop an RP-HPLC method for the simultaneous determination of cefpodoxime proxetil and clavulanic acid in pharmaceutical dosage forms. The present RP-HPLC method was validated following the ICH guidelines[7,8].

Acetonitrile HPLC grade, potassium dihydrogen orthophosphate and orthophosphoric acid AR grade were procured from Qualigens fine chemicals, Mumbai. Water HPLC grade was procured from S. D. Fine Chemicals. Reference standard of cefpodoxime proxetil and clavulanic acid were procured from Aurobindo Pharmaceuticals Ltd., Hyderabad, India and Cadila Pharmaceuticals Ltd., Ahmedabad, India.

Chromatographic separation was performed on a Shimadzu liquid chromatographic system equipped with a LC-10 VP solvent delivery system (Pump), SPD-10 vp UV/Vis detector, Rheodyne injector with 20 μl loop volume. Spinchrom software was applied for data collecting and processing. A Zorbax Eclipse XDB C_18_ column (150×4.6mm, 5 μ) was used for the separation. The mobile phase was a mixture of acetonitrile and 50 mM potassium dihydrogenphosphate buffer (pH 3.0 adjusted with 10% orthophosphoric acid) (70:30 v/v). It was filtered through a 0.2 μ membrane filter and degassed. A flow rate of 1.0 ml/min and a detection wavelength of 228 nm were used. Standard stock solutions of (1 mg/ml) of cefpodoxime proxetil, clavulanic acid and aspirin were prepared separately using a mixture of water and acetonitrile in the ratio 1:1 v/v. From the stock solution, mixed standard solution was prepared to contain 70 μg/ml of cefpodoxime proxetil, 20 μg/ml of clavulanic acid and 100 μg/ml of aspirin as internal standard. Twenty tablets, each containing 100 mg of cefpodoxime proxetil and 28.5 mg of clavulanic acid (CV-CEF 128, Bestochem Pharmaceuticals, Pondicherry, India) were weighed and finely powdered; a quantity of powder equivalent to 35 mg of cefpodoxime proxetil and 10 mg of clavulanic acid was weighed and transferred in to a 100 ml volumetric flask and the drugs were extracted with the mixture of acetonitrile and water (1:1 v/v) and volume was made up to 100 ml with the same solvent. The solution was filtered using Whatman filter paper and the filtrate is referred to as formulation solution. Two milliliter of this solution was pipetted in to a 10 ml volumetric flask containing 1 ml of aspirin (1000 μg/ml) as internal standard and this solution was used for the estimation.

With the optimized chromatographic conditions, a steady baseline was recorded. Twenty micro litres of the standard and formulation solutions were injected and the chromatogram was recorded (fig. 1). The retention time of clavulanic acid, cefpodoxime proxetil and aspirin was found to be 4.43, 6.4 and 5.61min, respectively. The response factor (peak area ratio of standard peak area and internal standard peak area) of the standard solution and sample solution were calculated. The concentration of drugs were calculated Table 1 using following formula, Concentration of drugs= (response factor of the sample/response factor of the standard)×concentration of standard. The peak area ratio of standard and sample solutions was calculated. The assay procedure was repeated six times and mean peak area ratio and mean weight of standard drugs were calculated. The percentage of individual drugs found in formulation, mean, standard deviation in formulation were calculated and presented in (Table 1). The results of analysis showed that the calculated amount of drug was in good agreement with the label claim of the formulation. The linearity of the method was determined at five concentration levels ranging from 70 to 350 μg/ml for cefpodoxime proxetil and 20 to 100 μg/ml for clavulanic acid. The calibration curve was constructed by plotting response factor against concentration of drugs. The slope and intercept value for calibration curve was y=0.0057x+0.0566 (R^2^ = 0.998) for cefpodoxime proxetil and y= 0.0023x+0.0628 (R^2^ = 0.999) for clavulanic acid. The results show that an excellent correlation exists between response factor and concentration range indicated above.

**Fig. 1:**
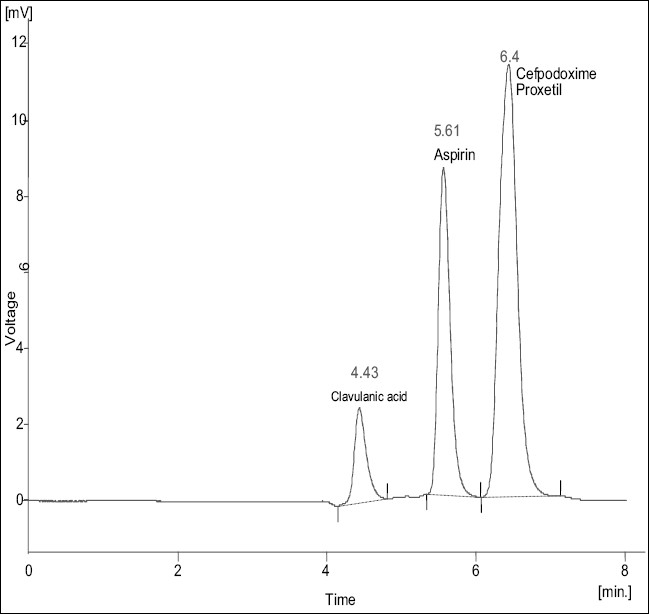
Typical chromatogram of sample solution Liquid chromatogram showing well resolved peaks of clavulanic acid (4.4 min), aspirin (internal standard, 5.6 min) and cefpodoxime proxetil (6.4 min) using mobile phase of acetonitrile:50 mM potassium dihydrogen phosphate buffer (pH 3.0) 70:30 v/v.

**TABLE 1:** RESULTS OF ANALYSIS OF FORMULATION AND RECOVERY STUDIES

Drug	Amount mg/tab		%Label claim*	% Recovery*
			
	Labelled	Found*		
Cefpodoxime proxetil	100	98.95	98.95±0.39	101.12
Clavulanic acid	28.5	29.18	102.42±0.52	100.23

* RSD of six observations

The method was validated as per ICH guidelines. The accuracy of the method was determined by recovery experiments. The recovery studies were carried out six times and the percentage recovery and standard deviation of the percentage recovery were calculated and are presented in Table 1. From the data obtained, added recoveries of standard drugs were found to be accurate.

The precision of the method was demonstrated by inter day and intra day variation studies. In the intraday studies, six repeated injections of standard and sample solutions were made and the response factor of drug peaks and percentage RSD were calculated. In the inter day variation studies, six repeated injections of standard and sample solutions were made for three consecutive days and response factor of drug peaks and percentage RSD were calculated. From the data obtained, the developed HPLC method was found to be precise. The limit of detection (LOD) and the limit of quantification (LOQ) of the developed method were determined by injecting progressively low concentrations of the standard solutions using the developed RP-HPLC method. The LOD is the smallest concentration of the analyte that gives a measurable response (signal to noise ratio of 3). The LOD for cefpodoxime proxetil and clavulanic acid was found to be 5 μg/ml and 3 μg/ml, respectively. The LOQ is the smallest concentration of the analyte, which gives response that can be accurately quantified (signal to noise ratio of 10). The LOQ for cefpodoxime proxetil and clavulanic acid was found to be 12 μg/ml and 10 μg/ml, respectively (Table 2).

**TABLE 2:** VALIDATION AND SYSTEM SUITABILITY STUDIES

Parameters	Cefpodoxime Proxetil	Clavulanic acid
Linearity range	70-350 μg/ml	20-100 μg/ml
Regression equation		y = 0.0023x+0.0628
Y= mx+c*	y =0.0057x+0.0566	
Correlation coefficient	0.9982	0.9993
Theoretical plates/meter	3146	3825
Resolution factor	2.0089	3.3636
Asymmetric factor	1.0	1.30
LOD (μg/ml)	5	3
LOQ (μg/ml)	12	10

* y=mx+c, m: slope c: y axis intercept

Ruggedness of the method was determined by carrying out the experiment on different time intervals and on different days. Robustness of the method was determined by making slight changes in the chromatographic conditions (Table 3). No marked changes in the chromatograms demonstrated that the HPLC method developed is rugged and robust. In order to demonstrate the stability of both standard and sample solutions during analysis, both solutions were analyzed over a period of 5 h at room temperature. The results show that for both solutions, the retention time and peak area of cefpodoxime proxetil and clavulanic acid remained almost unchanged (% RSD is less than 2) with no significant degradation within the period, indicated that both solutions were stable for at least 5 h, which was sufficient to complete the whole analytical process.

**TABLE 3:** ROBUSTNESS PARAMETERS

Parameter	Variation	Effect on chromatogram			
		
		Cefpodoxime proxetil		Clavulanicacid	
			
		Normal RT	After variation	Normal RT	After variation
Ratio of mobile phase					
	72:28	6.4	6.3	4.4	4.3
	68:32	6.4	6.4	4.4	4.5
Peak shape		sharp	sharp	sharp	sharp
Flow rate	1.2 ml	6.4	5.6	4.4	3.7
	0.8 ml	6.4	7.0	4.4	5.5
Peak shape		sharp	sharp	sharp	sharp
pH	3.2	6.4	6.2	4.4	4.2
	2.8	6.4	6.4	4.4	4.5
Peak Shape		sharp	sharp	sharp	sharp

RT = Retention time

The system suitability studies were carried out to determine theoretical plate/meter, resolution factor, asymmetric factor and tailing factor. The results are given in Table 2. The values obtained demonstrated the suitability of the system for the analysis of this drug combination, system suitability parameters may fall within ±3% standard deviation range during routine performance of the method. Overall, the proposed RP-HPLC method for the simultaneous estimation of cefpodoxime proxetil and clavulanic acid in combined dosage forms is accurate, precise, linear, robust, simple and rapid.
